# P-1347. Salmonella in a Teaching Hospital in the Dominican Republic: Clinical and microbiological characterization

**DOI:** 10.1093/ofid/ofaf695.1535

**Published:** 2026-01-11

**Authors:** Maltha Cruz-Abad, Anel E Guzmán-Marte, Ricardo Ernesto Hernandez-Landa, Osvaldo D Cabrera-Castellanos, Yeison Reyes-Burgos, Francisco Guzman-Ricardo, Ann S Sánchez-Marmolejos, Rita A Rojas-Fermín

**Affiliations:** Hospital Genral Plaza de la Salud, Santo Domingo, Distrito Nacional, Dominican Republic; Hospital General De La Plaza De La Salud, Distrito Nacional, Distrito Nacional, Dominican Republic; Universidad Ibero Americana, Santo Domingo, Distrito Nacional, Dominican Republic; Hospital General Plaza de la Salud, santo domingo, Distrito Nacional, Dominican Republic; Hospital General Plaza de la Salud, santo domingo, Distrito Nacional, Dominican Republic; Hospital General de la Plaza de la Salud, Santo Domingo, Distrito Nacional, Dominican Republic; Hospital General de la Plaza de la Salud, Santo Domingo, Distrito Nacional, Dominican Republic; Hospital General De La Plaza De La Salud, Distrito Nacional, Distrito Nacional, Dominican Republic

## Abstract

**Background:**

Fluoroquinolone-resistant *Salmonella* spp was classified by the WHO as a high priority pathogen due to its increasing resistance and high burden of infection, especially in low- and middle-income countries. This study describes the clinical and microbiological characteristics, comorbidities, and outcomes of patients with salmonellosis assisted in a teaching hospital.

**Figure:**
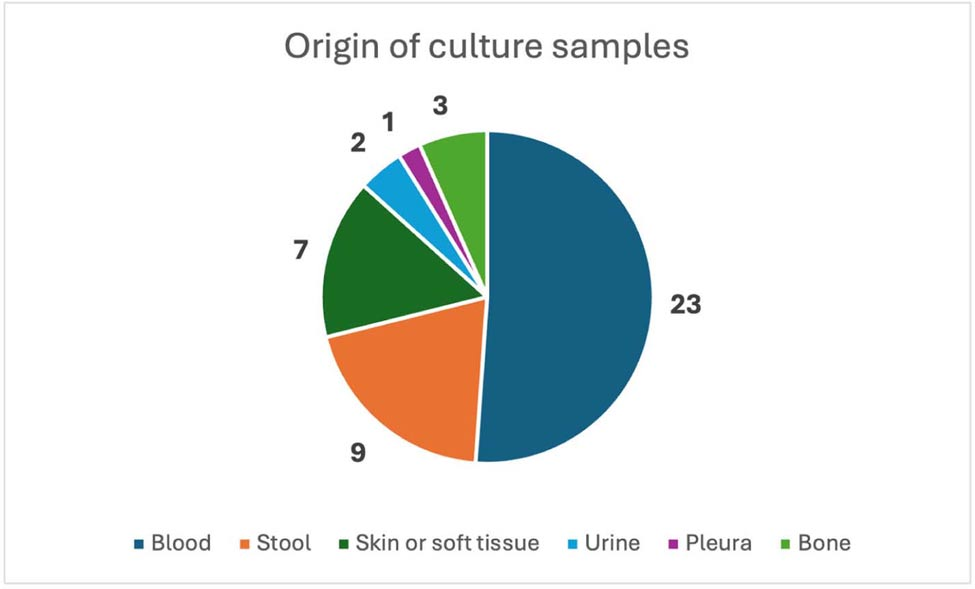
Origin of the salmonella samples

**Figure:**
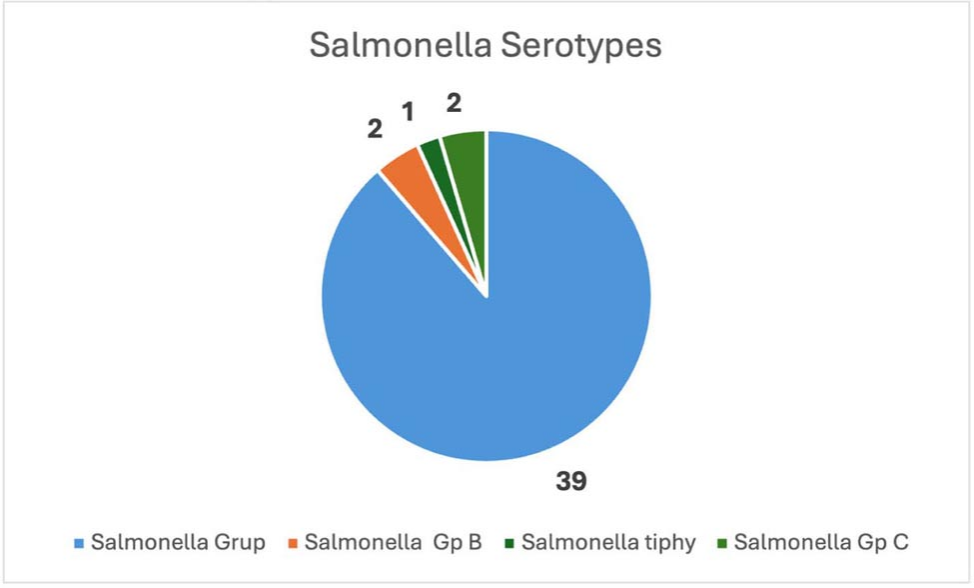
Salmonella Serotypes i

**Methods:**

We conducted a retrospective observational study including patients with a *Salmonella* spp. positive culture from April 12, 2021, to April 15, 2025. Antimicrobial susceptibility and resistance mechanisms were extracted from microbiology reports. Descriptive statistics and bivariate analysis were used to assess associations between clinical variables and mortality.

**Results:**

We included 44 isolates from 43 patients with a mean age of 41.3 ± 29.2 years (51.2% males). Mortality admission rate was 7/39 (17.9%). All deceased patients had positive blood cultures (7/23; 30.4%; p < 0.05) with a fluoroquinolone-resistant isolate. The most common comorbidities were hypertension (17/43), diabetes (7/43), neoplasia (7/43), and sickle cell disease (3/43) (2 of them had osteomyelitis). 39 of the *Salmonella* spp. isolates were not classified, 4 corresponded to non-typhoidal *Salmonella* (2 group B and 2 group C) and 1 was a *Salmonella typhi*. These isolates were cultured from blood (23), stool (9), skin or tissue (7), urine (3), and pleura (1). The non-susceptibility rate (intermediate or resistant) for ciprofloxacin was 92.7% (38/41), ceftriaxone 6.8% (3/44), cefepime 6.8% (3/44), and trimethoprim-sulfamethoxazole 4/21(19%). 3 isolates had a ESBL phenotype. Clinical presentations included Gastroenteritis (16/44) in which 7 only had blood culture positive, and 9 feces culture, skin and soft tissue infection (8/44), febrile syndrome (4/44) and community acquired pneumonia (5/44).

**Conclusion:**

This study highlights the impact of quinolone-resistant salmonella in low-medium income countries. There is a high mortality rate in hospitalized patients with salmonellosis, particularly in those with positive blood cultures, where fluroquinolones no longer represent an effective treatment option. We emphasize the need for rational antibiotic use and continuous surveillance to preserve available options.

**Disclosures:**

Rita A. Rojas-Fermín, MD, GSK: Honoraria

